# Mitochondrial Dysfunction Plays Central Role in Nonalcoholic Fatty Liver Disease

**DOI:** 10.3390/ijms23137280

**Published:** 2022-06-30

**Authors:** Raghu Ramanathan, Ahmad Hassan Ali, Jamal A. Ibdah

**Affiliations:** 1 Division of Gastroenterology and Hepatology, University of Missouri, Columbia, MO 65212, USA; raghu.ramanathan@health.missouri.edu (R.R.); aliah@health.missouri.edu (A.H.A.); 2 Harry S. Truman Memorial Veterans Medical Center, Columbia, MO 65201, USA; 3 Department of Medical Pharmacology and Physiology, University of Missouri, Columbia, MO 65212, USA

**Keywords:** liver, mitochondrial dysfunction, β-oxidation, nonalcoholic fatty liver disease, mitophagy

## Abstract

Nonalcoholic fatty liver disease (NAFLD) is a global pandemic that affects one-quarter of the world’s population. NAFLD includes a spectrum of progressive liver disease from steatosis to nonalcoholic steatohepatitis (NASH), fibrosis, and cirrhosis and can be complicated by hepatocellular carcinoma. It is strongly associated with metabolic syndromes, obesity, and type 2 diabetes, and it has been shown that metabolic dysregulation is central to its pathogenesis. Recently, it has been suggested that metabolic- (dysfunction) associated fatty liver disease (MAFLD) is a more appropriate term to describe the disease than NAFLD, which puts increased emphasis on the important role of metabolic dysfunction in its pathogenesis. There is strong evidence that mitochondrial dysfunction plays a significant role in the development and progression of NAFLD. Impaired mitochondrial fatty acid oxidation and, more recently, a reduction in mitochondrial quality, have been suggested to play a major role in NAFLD development and progression. In this review, we provide an overview of our current understanding of NAFLD and highlight how mitochondrial dysfunction contributes to its pathogenesis in both animal models and human subjects. Further we discuss evidence that the modification of mitochondrial function modulates NAFLD and that targeting mitochondria is a promising new avenue for drug development to treat NAFLD/NASH.

## 1. Introduction

Nonalcoholic fatty liver disease (NAFLD) is an umbrella term covering conditions characterized by excessive fat accumulation in the liver that are not triggered by alcohol intake. Excessive hepatic fat accumulation, which exceeds 5% of total liver weight, is a hallmark of NAFLD [1]. NAFLD is the most common chronic liver disease in the United States, affecting nearly 20–40% of the population [2,3], and has become among the top leading indications for liver transplantation in the United States [4,5,6,7]. Sedentary lifestyles, dietary changes, epidemic obesity, and type 2 diabetes have been identified as risk factors contributing to the global rise in NAFLD. Patients with NAFLD are at an increased risk of developing steatohepatitis (thus termed nonalcoholic steatohepatitis (NASH)) and fibrosis, which can eventually lead to cirrhosis and its consequent complications such as portal hypertension, progression to end-stage liver disease requiring liver transplantation, and the development of hepatocellular carcinoma (HCC) [8,9,10]. It is a multisystem illness affecting numerous extrahepatic organs, including insulin resistance, type 2 diabetes, and cardiovascular disease, all of which contribute to increased morbidity and mortality in patients with NAFLD/NASH [11,12]. The term metabolic- (dysfunction) associated fatty liver disease (MAFLD) has been recently proposed to more accurately reflect the disease’s heterogeneity and pathogenesis [13,14,15].

Multiple investigations have shown evidence of molecular, biochemical, and biophysical mitochondrial abnormalities in NAFLD; however, the exact mechanism(s) of mitochondrial dysfunction that leads to the development and progression of NAFLD remain unknown [16,17,18]. To date, there is no Food and Drug Administration (FDA)-approved drug therapies for NAFLD/NASH. There are several drug programs currently aiming to develop safe and effective medications for the treatment of NAFLD/NASH; one attractive approach is to correct the underlying mitochondrial abnormalities by replacing defective mitochondria with functional mitochondria. 

The objective of this review is to discuss our knowledge in understanding the central role of mitochondrial dysfunction in development and progression of NAFLD in both animal models and human subjects.

## 2. Nonalcoholic Fatty Liver Disease (NAFLD)

NAFLD is a chronic liver disease caused by the accumulation of excess fat in the liver in the absence of excessive alcohol intake [19]. It has a histological spectrum that ranges from simple steatosis to NASH (steatosis associated with lobular inflammation and hepatocellular ballooning), which can be complicated by fibrosis [20]. The prevalence of NAFLD is 4.6 times higher in obese subjects, with up to 74 percent having fatty liver [21]. It has been recently projected that the overall prevalence of NAFLD and NASH among individuals aged ≥15 years in the United States will be 33.5% and 27%, respectively, in the year 2030 [22,23]. About 5–8% of NASH patients progress to cirrhosis, an important risk factor for the development of HCC [23,24]. HCC is the second most common cause of cancer death in men and the sixth most common cause of cancer death in women worldwide [25]. Although NASH is a growing public health problem, only lifestyle modifications aimed at increasing physical activity and reducing caloric intake are currently recommended as treatments with limited success [26]. 

### 2.1. Epidemiology and Burden of NAFLD

NAFLD is the most prevalent cause of chronic liver disease worldwide, ranging from 13.5% in Africa to 31.8% in the Middle East, owing to disparities in overall caloric consumption, physical activity, body fat distribution, socioeconomic level, and genetic composition [27]. Type 2 diabetes is an important risk factor for NAFLD and is found in 45–65% of patients with type 2 diabetes and in up to 80% of individuals with obesity [28]. However, a healthy body mass index (BMI) does not seem to be protective against NAFLD, as non-obese subjects might develop NAFLD, generally referred to as lean NAFLD [29]. In addition, the economic burden of NASH is significant, with an estimated lifetime costs of NASH patients of USD 222.6 billion in the year 2017, and this is expected to rise in the coming decades [30]. Childhood obesity, which is a major risk factor for NAFLD, is on the rise; in the United States, the prevalence of obesity among children aged 2–5 years old grew from 84% in time period of 2011–2012 to 139% in the time period of 2015–2016 [31]. Taken together, these data underscore the alarming health and economic impact of NASH and call for immediate actions.

### 2.2. Pathogenesis of NAFLD

Different theories have been proposed in recent years to help explain the development and progression of NAFLD. The “multiple-hit” hypothesis in NASH has replaced the “two-hit” one, which considers multiple injuries in genetically susceptible individuals, including insulin resistance, hormones secreted from the adipose tissue, gut microbiota, and nutritional factors [32,33]. The excess calories from food are stored in the form of fat in adipose tissue as triglycerides [34]. Hepatic free fatty acids (FFAs) and triglycerides (TGs) accumulate as a result of lipid metabolism dysregulation [35]. At least three mechanisms have been identified as sources of excessive lipid accumulation in the liver from a pathological perspective: enhanced visceral adipose tissue (AT) lipolysis, hepatic de novo lipogenesis (DNL) activation, and a high-calorie and/or -fat diet [36]. Excess calorie intake has been proposed to create an imbalance in gut microbiota, leading to increased bacterial products in the portal circulation and activating the innate immune system, thus leading to NAFLD/NASH [37,38]. Due to increased circulating plasma FFA, the body develops insulin resistance in the muscle, which is a major step in the development of NAFLD [39,40]. Insulin resistance in turn increases hepatic DNL, causes the secretion of adipokines and inflammatory cytokines via the adipose tissue, and generates an imbalance in adipose tissue lipolysis [41]. Simultaneously, hepatic fat accumulation causes lipotoxicity, a condition that promotes oxidative stress and exerts a deleterious effect on mitochondrial and cellular function [42].

**Figure 1:**
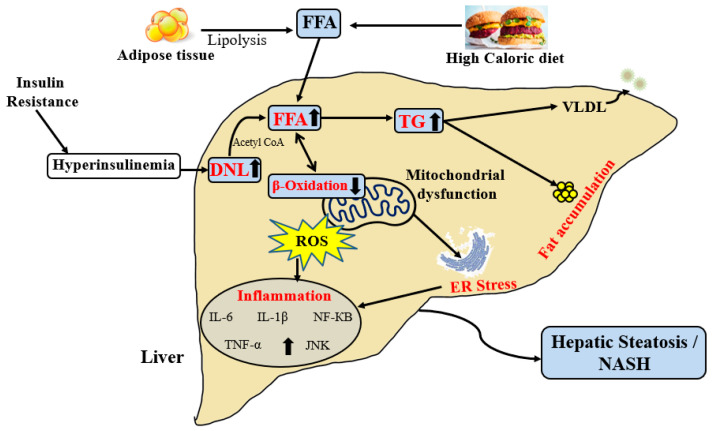
Pathogenesis of NAFLD. Enhanced visceral adipose tissue lipolysis, hepatic DNL, and high-caloric diet contribute to lipid accumulation in the liver. NAFLD: non-alcoholic fatty liver disease; FFA: free fatty acid; VLDL: Very low-density lipoprotein; DNL: de novo lipogenesis; ER: endoplasmic reticulum; ROS: reactive oxygen species; TNF-α: tumor necrosis factor-α; NF-KB: nuclear factor-KB, IL: Interleukin; JNK: c-jun N terminal kinase.

Different sources of fatty acids contribute to hepatic lipid accumulation in NAFLD [36]. As shown in Figure 1, the FFAs used for TG synthesis in hepatocytes come from a variety of sources, including dietary supply, lipolysis, and DNL [43]. Nearly 59% of the fatty acids stored in the liver originate from adipose tissue lipolysis, 26% originate from DNL, and 15% originate from the diet [36]. FFAs can be converted into TGs and exported as very low-density lipoproteins (VLDLs) [44]. As a result of disturbing the normal physiological balance, hepatic lipid accumulation leads to NAFLD (Figure 1). The increase in circulating FFA and TG levels along with the storage of TG in the adipose tissue causes peripheral insulin resistance (IR). Obese patients gradually develop type 2 diabetes and fatty liver due to successive hyperglycemia and compensatory hyperinsulinemia. Continuous adipocyte lipolysis associated with IR leads to elevated levels of circulating FFAs, which are readily transported to the liver. Additionally, high carbohydrate consumption increases hepatic de novo lipogenesis, which causes increased FFA synthesis and uptake, resulting in fatty liver [45]. It has been shown that patients with NAFLD may have abnormally high levels of hepatic DNL [46]. SREBP1c (sterol regulatory element-binding protein 1c), which regulates hepatic DNL, can be induced by a high-carbohydrate diet [47]. Recent studies have shown that NAFLD patients have higher expression of SREBP1c [48]. In addition, mitochondrial β-oxidation, which is the primary pathway for fatty acid oxidation, is seriously affected in NAFLD [49]. Malonyl CoA is the major substrate of DNL and also inhibits carnitine palmitoyl transferase 1 (CPT-1). It has been shown that both elevated DNL and defective β-oxidation boost lipid accumulation [50]. Koliaki et al. have shown that increased lipid availability in the liver of obese humans with NAFLD initially stimulates the hepatic mitochondrial capacity; however, it ultimately promotes excessive hepatic oxidative stress and reduces mitochondrial functionality, leading to the progression of NAFLD to NASH [51]. Our group has previously reported that hepatic mitochondrial dysfunction precedes the onset of NAFLD and insulin resistance in an obese rodent model [52].

Increased FFA levels in the hepatocytes can activate inflammatory pathways, leading to liver injury [53]. Further, the alternative peroxisomal and cytochrome oxidation of FFA occurs when mitochondrial β-oxidation is impaired, resulting in significant levels of reactive oxygen species (ROS) and hazardous by-products [54,55]. In addition, the endoplasmic reticulum (ER) plays an important role in stress response by mediating a specific set of intracellular pathways, collectively known as the unfolded protein response [56,57,58]. Reports also suggest that there is continuous inter-organ cross talk involved in NAFLD pathogenesis, with the adipose tissue and the gut playing key roles [59]. NAFLD pathogenesis is also influenced by genetic and epigenetic factors, with heritability estimates ranging from 20 to 70% [60,61]. Several genome-wide studies have reported that the existence of multiple single-nucleotide polymorphisms in genes such as patatin-like phospholipase domain-containing protein-3 (PNPLA3) is independently correlated with the development and severity of NAFLD as well as an increased risk of HCC in NAFLD patients [62].

### 2.3. Diagnosis of NAFLD

NAFLD is asymptomatic in nature and mostly diagnosed in patients who come for routine laboratory checkups due to other co-morbid disease with elevated transaminase serum levels, which tend to decline as the pathology progresses from fibrosis to cirrhosis [63]. The European Association for the Study of the Liver, the European Association for the Study of Diabetes, and the European Association for the Study of Obesity (EASL-EASD-EASO) recommend imaging techniques, particularly ultrasonography (US), to diagnose NAFLD because it is more widely used and less expensive than the gold standard, magnetic resonance imaging (MRI) [64]. In cases of moderate and severe steatosis, the above approaches are regarded as first-line diagnostic testing, as they also provide information on the hepatobiliary system. Recent studies in animal models have found that liver autofluorescence (AF) spectroscopy could be useful for both the experimental and clinical early diagnosis of NAFLD/NASH [65]. The gold standard for diagnosing NASH and staging hepatic fibrosis in individuals with NAFLD is liver biopsy, although sampling variability can be a limitation [66].

Several non-invasive cross-validated scoring approaches, such as the NAFLD fibrosis score (NFS), fibrosis-4 (FIB-4) index, and aspartate aminotransferase-to-platelet ratio index (APRI), are commonly used in clinical practice for the assessment of the stage of fibrosis in patients with NAFLD/NASH [67]. The Enhanced Liver Fibrosis (ELF) score is a blood biomarker that is commonly utilized in clinical trials [68]. Pro-C3 is a biomarker for fibrogenesis that measures the propeptide cleaved from the intact collagen molecule [69]. The use of ultrasound-based transient elastography (Fibro-Scan) to evaluate liver stiffness is another technique used for the assessment of liver fibrosis in NAFLD patients [70,71,72].

### 2.4. Management of NAFLD

Treatment for patients with NAFLD comprises lifestyle and diet recommendations, with a focus on weight loss and physical activity. A landmark study reported that weight loss of at least 5–7% is required to alleviate hepatic steatosis, but about 8–10% weight loss is required to improve inflammation and fibrosis in patients with NASH [73]. Bariatric surgery has been shown to improve steatosis, inflammation, and regress fibrosis in patients with obesity complicated by NASH [74]. However, bariatric surgery is not considered a first-line therapy for the treatment of NASH [75]. 

#### 2.4.1. Diet Restriction

NAFLD is linked to a high-calorie and high-carbohydrate diet [76]. Dietary adjustments as well as caloric restriction and weight loss are the pillars for NAFLD treatment. It is recommended to ingest a high-quality healthy diet that is low in saturated fat and high in monounsaturated and polyunsaturated fatty acids (MUFA) and complex carbohydrates [77]. The Mediterranean diet is among the recommended diets, and it includes vegetables, fruit, nuts, olive oil, and seafood and avoids red meat, processed foods, and refined sugar [78].

#### 2.4.2. Weight Loss

In order to improve NAFLD, a hypocaloric diet that results in weight loss is required. Significant improvement in liver fibrosis and inflammation in NASH have been linked to weight loss of 10% or more [73]. Weight loss should be gradual and can be accomplished with a hypocaloric diet and exercise [73]. However, caution should be exercised when implementing these lifestyle measures because rapid weight loss has been reported to enhance liver inflammation and fibrosis [79,80]. Intermittent fasting has been reported to assist in weight loss [81].

#### 2.4.3. Exercise

Sedentary lifestyles, such as increased hours of television watching or working on-a computer for longer periods of time, raise the risk of NAFLD [82]. Exercise, on the other hand, is beneficial to one’s health, and studies have shown that regular exercise in NAFLD patients reduces body weight, insulin resistance, and the degree of fatty liver [83]. In NAFLD patients, aerobic training has been shown to lower serum levels of alanine aminotransferase (ALT) and aspartate aminotransferase (AST), which are liver injury biomarkers [84]. Previously published reports from our group have shown that daily exercise with caloric restriction prevents NAFLD in rats by improving hepatic fatty acid oxidation and mitochondrial function [85,86]. 

#### 2.4.4. Pharmacological Interventions

Currently, there are no FDA-approved drugs for the treatment of NAFLD. Various pharmacological approaches for treating NAFLD have shown some success. Metformin improves hepatic and peripheral insulin resistance by lowering glucose absorption, boosting β-oxidation, and decreasing hepatic gluconeogenesis and FA production [87]. Metformin treatment improves cholesterol and aminotransferase levels in patients with NAFLD [88]. Peroxisome proliferator-activated receptor (PPAR)-γ agonists, such as pioglitazone and rosiglitazone, have been shown to have beneficial effects in patients with NAFLD [89]. A landmark randomized clinical trial has found that pioglitazone dramatically reduces steatosis, inflammation, hepatocellular ballooning, and fibrosis [90]. In NASH patients, therapeutic medications that address oxidative stress and inflammation, as vitamin E, have also been used [90]. Vitamin E has been shown to be beneficial, particularly in children with mild NAFLD/NASH [91]. Hydrophilic bile acids (Bas) are another pharmacological approach for the treatment of NASH, as they can control both glucose and lipid metabolism via activating the farnesoid X receptor (FXR) [92].

## 3. Mitochondria and NAFLD

Mitochondria are highly dynamic organelles that serve as the center for energy metabolism. The liver consumes about 15% of the organism’s oxygen under normal conditions, implying that hepatocytes are rich in mitochondria, which require oxygen to make adenosine triphosphate (ATP) [93]. Mitochondria make up to 18% of the total volume of a hepatocytes and play a critical role in the liver’s metabolic functions as well as nutrient (carbohydrates, lipids, and proteins) oxidation for energy generation [94]. The balance between mitochondrial biogenesis, mitochondrial fission/fusion, and mitochondrial autophagy is essential for mitochondrial homeostasis. Emerging evidence suggests that cellular functions are at risk when mitochondrial homeostasis is disrupted [95,96].

### 3.1. Mitochondrial Fatty Acid Oxidation

Mitochondria are the cells’ “powerhouse”, as they are the primary source of energy. Inside mitochondria, dietary glucose and fatty acids are oxidized through β-oxidation and the tricarboxylic acid (TCA) cycle. In fatty acid catabolism, FFAs are converted to fatty acyl-CoA in the hepatocyte cytosol, which then enters into mitochondria via carnitine palmitoyl-transferase 1 (CPT1). The first step of β-oxidation of long-chain fatty acids is the dehydrogenation of the acyl-CoA easter by acyl-CoA dehydrogenases followed by the three steps carried out by mitochondrial trifunctional protein (MTP). The β-oxidation breaks the FFA into acetyl CoA, which can be degraded to CO_2_ and H_2_O by the TCA cycle. The electron transport chain (ETC), which is found in the inner mitochondrial membrane, is the main site of ATP production. It is made up of five complexes numbered from I to V. This aerobic process necessitates the presence of a reducing component, which is provided by the electron carriers NADH and FADH_2_. Electron transfer occurs from complex I to complex IV by providing energy to pump protons from the mitochondrial matrix to the intermembranous region, which will lead to a proton gradient. The potential energy in this gradient is then used by complex V for the synthesis of ATP.

### 3.2. Mitochondrial Dynamics, Biogenesis and Mitophagy

Mitochondrial dynamics involve the fusion and fission of mitochondria [97]. Under the conditions of metabolic or environmental stress, mitochondrial fission and fusion processes are critical for maintaining the function of the mitochondria. Fusion is a process in which the contents of partially damaged mitochondria are mixed to promote complementation, which helps to mitigate cellular stress. Fission is a process that leads to the creation of new mitochondria, which also contributes to quality control by separating the damaged mitochondria from the healthy mitochondria [98,99]. Disruptions of these processes have been implicated in disease. In mammalian cells, mitofusin-1 (Mfn1) and mitofusin-2 (Mfn2) are responsible for the fusion process of the outer mitochondrial membrane [100], while the optic atrophy 1 (Opa1) protein regulates the fusion of the inner mitochondrial membrane [101,102]. The primary proteins involved in fission are the dynamin-related protein 1 (Drp1) and Mitochondrial Fission 1 Protein (Fis1) [103,104]. In mitochondrial bioenergetics, the balance between fusion and fission processes is critical. Intracellular stress and external factors can disrupt the balance between fusion and fission, resulting in mitochondrial fragmentation [105]. Excessive fission is characterized by increased levels of the fission protein dynamin-related protein 1 (Drp1). Mitochondrial dysfunction is associated with the dysregulation of the proteins involved in mitochondrial fission [106,107].

Mitochondrial biogenesis comprises mitochondrial DNA (mtDNA) replication, the transcription of mtDNA and nuclear coding genes, and the translation and assembly of the OXPHOS complex, all of which are important for cellular homeostasis and survival [108]. Peroxisome proliferator-activated receptor gamma coactivator-1 (PGC-1α) is a master regulator of mitochondrial biogenesis and a major transcriptional activator [109]. It regulates mitochondrial biogenesis by regulating the expression of nuclear and mitochondrial genes by activating a number of other transcription factors such as nuclear respiratory factors 1 and 2 (NRF-1 and NRF-2), resulting in the expression of mitochondrial transcription factor A (TFAM) [110,111]. TFAM interacts directly with the mitochondrial genome and mitochondrial transcription factors, resulting in the transcription of mitochondrial genes [112]. Furthermore, PGC-1α increases mitochondrial FAO by acting as a co-activator of peroxisome proliferator-activated receptor α and γ (PPARα and PPARγ), which leads to the production of mitochondrial β-oxidation genes [113,114]. Moreover, PGC-1α activation stimulates mitochondrial biogenesis as manifested by the stimulation of mitochondrial DNA replication and mitochondria gene expression [115,116]. Through the phosphorylation of PGC-1α, AMP-activated protein kinase (AMPK) is also involved in the regulation of mitochondrial biogenesis [117]. 

Mitophagy is a mitochondrial-specific autophagy that involves the selective isolation and degradation of damaged mitochondria to maintain the functional integrity and cellular homeostasis [118]. The PTEN-induced kinase 1 (PINK1)—Parkin pathway, plays an important role during mitophagy [119]. Mitophagy is a protective mechanism enabling the cell to avoid generating reactive oxygen species (ROS) due to damaged mitochondria and maintains redox balance [120]. In addition, mitophagy also stimulates lipid droplet breakdown and the release of FFAs, which are then transported to healthy mitochondria for β-oxidation and increased energy release.

### 3.3. Role of Mitochondrial Dysfunction in NAFLD

Mitochondrial dysfunction and oxidative stress have been observed in liver tissue from patients with fatty liver disease [121]. It is characterized by various degrees of ultrastructural mitochondrial damage, abnormal morphologic changes, respiratory chain activity reduction, ATP depletion, increased permeability of the outer and inner membranes, ROS overproduction, oxidative stress-mediated deletions of mtDNA, and impaired mitochondrial β-oxidation [50,122]. Several recent investigations have suggested that mitochondrial dysfunction is a major factor in the development and progression of NAFLD [123]. The exact mechanism by which mitochondrial dysfunction contributes to NAFLD is still not fully understood. Reduced FAO, increased FFA delivery and transport into the liver, and increased hepatic fatty acid synthesis have been reported to be involved in the pathogenesis of NAFLD [121]. 

#### 3.3.1. FAO and NAFLD

Mitochondrial dysfunction has been linked to a reduction in the β-oxidation of lipids, resulting in triglyceride accumulation in hepatocytes [124]. In humans with obesity and NAFLD, a moderately malfunctioning hepatocyte ETC has been reported [51]. High amounts of malonyl-CoA have been shown to inhibit CPT-1, leading to a reduction in β-oxidation [121]. Acylcarnitine accumulation has also been reported as a marker of mitochondrial stress, malfunction, and reduced FAO [125]. Impaired β-oxidation influences peroxisomal and cytochrome oxidation of FFAs, resulting in significant levels of ROS and hazardous byproducts [54]. Our group has previously reported that defects in mitochondrial FAO induce hepatic steatosis in mice without progressing to cirrhosis [126,127]. Human MTP defects are recessively inherited, and children who are deficient in one or more of the three enzymatic functions in MTP have been found to have microvesicular hepatic steatosis [128]. Our previous research has shown that homozygous mice with MTP deficiency develop hepatic steatosis shortly after birth [129]. Further, we previously reported that aged mice heterozygous for MTP abnormalities develop insulin resistance and hepatic steatosis [126]. Finally, we observed that MTP modulation rescued NAFLD in mice [130]. 

More recently, our group has investigated the relationship between NAFLD/NASH, mitochondrial fatty acid oxidation in liver, and hepatic mitochondrial quality in human subjects [131]. We obtained liver biopsies from patients with obesity undergoing bariatric surgery and correlated the liver histology to the mitochondrial fatty acid oxidation measured in the liver tissue. The results of this study showed that hepatic mitochondrial complete FAO was reduced by ~40–50% in subjects with NASH compared to the control subjects with normal histology. This decrease corresponded with increased hepatic mitochondrial reactive oxygen species production and reductions in the markers of mitochondrial biogenesis and mitophagy [131]. These findings support a link between mitochondrial dysfunction and NAFLD/NASH in humans and suggest that impaired hepatic fatty acid oxidation and reduced mitochondrial quality are strongly linked to increasing NAFLD severity in patients with obesity. Further, in our ongoing study in veterans undergoing liver biopsy for suspected NASH, measurement of the FAO in the liver tissue showed that hepatic FAO was ~40% lower in veterans with liver fibrosis compared to those without fibrosis (unpublished data recently presented in abstract format at the American Association for the Study of Liver Diseases (AASLD) annual meeting [132]). There were no significant differences in the FAO among various fibrosis stages. These findings suggest that compromised hepatic mitochondrial fatty acid oxidation is linked to NAFLD progression and fibrosis development in the veteran population. Our human studies are the first to directly measure mitochondrial FAO and mitochondrial quality in liver tissue obtained from NAFLD/NASH patients. 

Mitochondrial dysfunction results from oxidative mitochondrial damage, with abnormalities in the mitochondrial ETC and OXPHOS [133]. NAFLD patients have been found to have a higher rate of mtDNA mutations, including genes encoding ETC complexes, with the mutational burden increasing with the severity of the histopathological abnormalities [134]. Reduced expression of their corresponding messenger RNA was linked to mutations in genes encoding ETC subunits [134]. Previous studies have reported that in the liver tissue of patients and animal models with NAFLD, the mitochondrial respiratory chain complex is reduced [125,135]. Compared to individuals without NAFLD, patients with NAFLD have been found to have a reduction in respiratory chain activity of 37% in complex I, 42% in complex II, 30% in complex III, 38% in complex IV, and 58% in complex V [125]. According to a previous report, complex I and IV subunits were drastically reduced in CDD-fed mice [136].

#### 3.3.2. Insulin Resistance and NAFLD

Insulin resistance (IR) has been linked to reduced mitochondrial numbers, abnormal mitochondrial morphology, decreased levels of mitochondrial oxidative enzymes, and decreased ATP production both in vivo [137,138] and ex vivo in human muscle biopsies [139]. Increased intracellular lipid levels caused by elevated FFA levels in the plasma are linked to insulin resistance in the liver and muscle [140]. In adipocytes from type 2 diabetic patients or morbidly obese human beings, there are significantly less mitochondria, and fewer genes that are expressed during mitochondrial biogenesis [141]. Hence, IR metabolic tissues, such as skeletal muscle, liver, and fat, frequently exhibit a decreased mitochondrial number, decreased mitochondrial gene expression, abnormal mitochondrial morphology, and abnormal activities in oxidative phosphorylation. These mitochondrial abnormalities are linked to insulin resistance, intracellular lipid synthesis, and the pathogenesis of type 2 diabetes and NAFLD. Finally, insulin resistance is caused by mitochondrial dysfunction, which increases ROS levels. These elevated ROS activate many serine kinases, which phosphorylate IRS proteins [142]. As a result, increased ROS production brought on by lipid-induced mitochondrial dysfunction reduces insulin signaling both directly and indirectly. The angiopoietin-like protein 4 (ANGPTL4), which is released by the liver and adipose tissue [143], is crucial for controlling the metabolism of lipids. Although ANGPTL4 has been linked to lipid homeostasis [144], there is still debate on how it affects glucose metabolism [145]. ANGPTL4 was found to promote lipolysis [146] and to prevent the removal of triglycerides from plasma by inhibiting lipoprotein lipase [147]. According to recent studies, mice who completed treadmill exercise also showed increased ANGPTL4 mRNA expression in the liver [148].

In IR individuals and animal models, exercise increases insulin action and glucose tolerance [149]. Significant evidence suggests that aerobic exercise promotes mitochondrial biogenesis by elevating PGC-1α, NRF-1, and TFAM gene expression. In order to improve whole-body glucose metabolism, endurance exercise training increases mitochondrial size, number, and oxidative activity [150]. Skeletal muscle electron transport chain activity and mitochondrial cristae are increased by moderate-intensity exercise and weight loss, improving insulin sensitivity [151]. Aerobic exercise restores the age-related decline in mitochondrial gene expression and mitochondrial biogenesis [152]. Exercise therefore enhances glucose and lipid metabolism by AMPK and PGC-1 activation, which increases mitochondrial biogenesis and function.

#### 3.3.3. ROS and NAFLD

Excess ROS production in NAFLD has been reported to be linked to ETC disruption, permeabilization of the outer mitochondrial membrane, altered mitochondrial membrane potential (Δ_Ψm_), and alterations in mitochondrial structural integrity [52]. Excess ROS has been shown to induce oxidative base lesions in mtDNA bases, which has been shown to induce apoptosis [153,154,155]. Mitochondrial DNA mutations caused by oxidative modifications are damaging to cellular integrity and are linked to higher ROS leakage from organelles and cells [156]. The initial cause of this ROS-damaging cycle is believed to be long-chain FFAs, which accumulate in nonalcoholic fatty liver [157]. It has been previously reported that NAFLD is characterized by mtDNA depletion and elevated liver levels of 8-hydroxy-2’-deoxyguanosine (8-OHdG), a DNA oxidation marker [51]. We previously observed that ultrastructural abnormalities in the mitochondria of an NAFLD rat model, such as cristae disruption, hypodense matrix, and swelling/rounding, were strongly associated with enhanced ROS generation [52]. When ROS stimulates NF-κB and the nuclear-binding oligomerization domain-like receptor family and the pyrin domain-containing 3 (NLRP3) inflammasome, inflammatory cytokines such as IL-1β, IL-6, and TNF-α are generated [158]. Mitochondrial permeability transition pores (MPTP), which release mtDNA into the cytoplasm and serve as a danger-associated molecular pattern (DAMP), can arise as a result of mitochondrial membrane disruption caused by ROS. The NLRP3 inflammasome is subsequently activated by MPTP, which causes the cytokine IL-1β to mature and prolong inflammation [159]. These proinflammatory cytokines also activate Kupffer cells, which produce proinflammatory cytokines that increase the inflammatory response in the liver. As a result of MPTP synthesis, immunological mediator cells such as Toll-like receptor 9 (TLR9) and formyl peptide receptor 1 (FPR1) are activated, leading to hepatocyte necrosis and the extracellular release of mitochondrial substances such as mitochondrial DNA and N-formyl peptide [160,161,162].

In the livers of rodents fed HFD or choline-deficient diets or an ethionine-supplemented (CDE) diet, PGC-1α and mitochondrial biogenesis were suppressed along with TFAM downregulation [163,164]. Sirt1 overexpression in the nucleus and Sirt3 overexpression in the mitochondria have been demonstrated to improve antioxidant defense enzymes and decrease proinflammatory pathways [165], providing protection against NAFLD and acting as sensors to promote PGC-1α expression. Sirt3 has been found to inhibit the adaptive response to high levels of hepatic FFA, resulting in mitochondrial dysfunction [166,167]. As a result, in the early stages of hepatocyte steatosis, primary mitochondrial dysfunction activates multiple adaptive metabolic pathways to reduce oxidative damage, which are mediated by increased mitochondrial activity and the increased expression of ROS detoxification genes by PGC-1α as well as Sirt1 and Sirt3 [168]. The PGC-α1-NRF-2 complex causes mitochondrial transcription factors B1 and B2 (tfb1m and tfb2m) to be expressed in mice [169]. Further, mitophagy is increased, and mitochondrial mass as well as mtDNA and PGC-1α expression are decreased in advanced NAFLD, all of which contribute to a vicious cycle of hepatic mitochondrial depletion and dysfunction. Deficient regulation of hepatocyte mitophagy promotes enhanced oxidative stress and inflammation in the late versus early stages of NAFLD [170,171].

**Figure 2:**
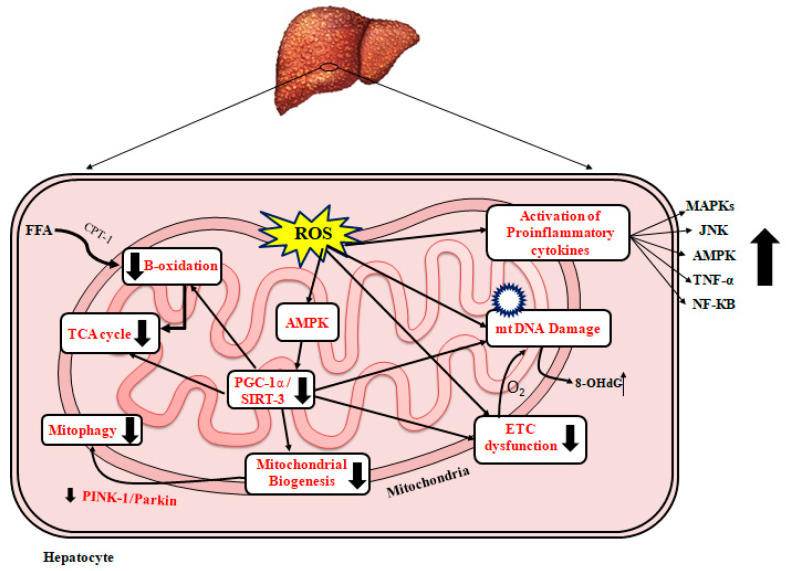
Mitochondrial dysfunction and NAFLD. A schematic diagram representing the effect of oxidative stress leading to mitochondrial dysfunction in liver leading to NAFLD. FFA: free fatty acid; TCA: tricarboxylic acid cycle; ROS: reactive oxygen species; CPT-1: carnitine palmitoyl transferase 1; AMPK: AMP-activated protein kinase; PGC-1α: peroxisome proliferator-activated receptor-alpha; ETC: electron transport chain; SIRT3: sirtuin 3; MAPKs: mitogen-activated protein kinase; TNF-α: tumor necrosis factor-α; NF-KB: nuclear factor-KB, IL: Interleukin; JNK: c-jun N terminal kinase; 8-hydroxydeoxyguanosine; O_2_: oxygen; Pink: PTEN-induced kinase 1.

Patients with NASH have been found to have ultrastructurally damaged mitochondria in their liver tissue [155,172]. In addition, higher amounts of ROS and ROS-mediated mtDNA damage have been described in patients with NASH compared to those without NASH [51,173]. Increased ROS levels have been reported to directly promote inflammation by activating inflammatory signaling pathways such as the NF-κB and JNK pathways as well as indirectly by increasing the gene expression of inflammatory cytokines such as TNF-α, TGF-β, and Fas ligand [174]. TNF-α has been found to stimulate the release of cytochrome C from the mitochondria and leak into the cytosol, causing both apoptosis and necrosis [175]. Figure 2 represents a schematic diagram of the effects of ROS leading to mitochondrial dysfunction and the development/progression of NAFLD, mitochondrial biogenesis, and NAFLD

#### 3.3.4. Mitochondrial Biogenesis and NAFLD

The mitochondrial quality control (MQC) mechanism involves the complex regulation of several processes such as biogenesis, dynamics, and mitophagy, all of which are important for maintaining cellular homeostasis. The failure of the quality control processes leads to mitochondrial dysfunction [176,177]. AMPK is a key regulator of fatty acid metabolism in the liver with downstream mediators such as sirtuins (SIRTs) and PGC-1α [178]. SIRTs are a family of NAD-dependent protein deacetylases, with the SIRT3 residing in the mitochondrial matrix being the most studied. Under normal physiological conditions, SIRT3 and PGC-1α can regulate each other [167]. SIRT3 activates many targets, including β-oxidation enzymes and AceCS (acetyl-CoA synthase) for acetyl-CoA formation [179]. SIRT3 controls ATP synthesis by regulating respiratory chain complex I and II [180]. In addition, SIRT3 has been found to prevent oxidative damage to the mitochondria by interacting with essential antioxidant enzymes including superoxide dismutase 2 (SOD2) and isocitrate dehydrogenase 2 (IDH2) [181,182]. PGC-1α regulates mitochondrial biogenesis and is essential for its function. PGC-1α activates the expression of genes involved in β-oxidation, the TCA cycle, OXPHOS, and mtDNA replication by acting as a coactivator of nuclear receptors and nuclear transcription factors such as PPARs, estrogen-related receptors (ERRs), and NRFs [183]. In addition to AMPK, upstream mediators of PGC-1α include cGMP, endothelial NO synthase, and exogenous NO, all of which have been found to increase mitochondrial biogenesis when PGC-1α is activated [184,185,186,187]. In NAFLD, hepatocytes have been found to have low AMPK activity, which causes mitochondrial dysfunction [188]. PGC-1α mRNA expression in the liver has been found to be diminished in NAFLD patients, resulting in decreased hepatic mitochondrial respiration [51]. Furthermore, SIRTs are downregulated in the livers of NAFLD patients, causing the hyperacetylation of various mitochondrial proteins and impairing mitochondrial function [189]. Increased SIRT3 expression in hepatocytes has been found to increase mitochondrial respiratory ability and redox homeostasis, reducing both hepatic lipid accumulation and oxidative stress [190]. Recently, our group has reported that in vivo SIRT3 overexpression in a mouse model heterozygous for an MTP defect resulted in a significant increase in mitochondrial fatty acid oxidation with increased hepatic levels of MTP. Further, we have demonstrated that SIRT3 overexpression rescued NAFLD in MTP heterozygous mice fed a high-fat diet [130]. 

#### 3.3.5. Mitophagy and NAFLD

Mitophagy causes the removal of overly damaged mitochondria and protects against the progression of NAFLD by oxidative stress [191,192]. Changes in mitophagy has been found to cause the accumulation of severely damaged and defective mitochondria, resulting in cell necrosis [193]. In a NASH mouse model fed a Western diet for more than 2 months, Fis1 and Drp1 protein levels were reported to be decreased, which was followed by hepatic inflammation and liver fibrosis [194]. Drp1-related mitochondrial fission has been found to be induced by a high-fat diet, which lowers levels of anti-inflammatory cytokines, including interleukin-10 (IL-10) and IL-13, implying a role for mitochondrial fission in hepatic inflammation [195]. Furthermore, the SIRT1/SIRT3-FOXO3a signaling pathway regulates Drp1 transcript levels [196]. Mfn2 levels have been found to be decreased in liver samples from NASH patients and in mouse models of steatosis and NASH; Mfn2 re-expression has been reported to improve disease in NASH mouse models, while the liver-specific deletion of Mfn2 causes inflammation, triglyceride accumulation, fibrosis, and liver cancer [197]. 

Mitophagy has been proposed as a key player in NAFLD. The liver mitochondria of Bnip3-deficient mice have been found to have a lower Δ_Ψm_, abnormal shape, and reduced oxygen consumption, which has been found to be associated with increased ROS, inflammation, and steatohepatitis-like histological abnormalities [198]. The expression of PINK1 and Parkin has been found to be downregulated and associated with the activation of the mitochondria-related apoptotic pathway and mPTP opening in a high-fat-fed NAFLD rat model [199].

#### 3.3.6. Targeting Mitochondria for Treatment of NAFLD

To date, lifestyle modifications such as dietary intervention and exercise remain the basis of NAFLD treatment, with limited success. Currently, there are no FDA approved medications for the treatment of NAFLD/NASH. Considering that mitochondrial dysfunction plays a central role in the development and progression of NAFLD, targeting mitochondria and the modulation of mitochondrial function is a promising field for the treatment of NAFLD. One dietary pattern that has acquired acceptance among the general public as a successful weight-loss method is the ketogenic diet, which is composed of a high-fat, moderate-protein, and low-carbohydrate diet and can lead to weight loss and an improvement in glycemic control, but it also carries some risk [200]. A recent study suggests that the hepatic mitochondrial fluxes and redox status of humans are altered by the ketogenic diet, which effectively treats NAFLD [201]. Our group has recently demonstrated that mitochondrial FAO modifications can modulate NAFLD [130]. We have reported that SIRT3 overexpression increases hepatic levels of MTP, increases mitochondrial FAO, and rescues NAFLD in MTP heterozygous mice fed a high-fat diet for 16 weeks [130]. In a recent study, antioxidant foods such as blueberry juice have been shown to significantly reduce NAFLD-induced mitochondrial swelling and hepatic necrosis by restoring mitochondrial respiratory chain function and suppressing ROS production [202]. 

Melatonin has been demonstrated to improve mitochondrial ROS, mitochondrial fission, and mitophagy inhibition in NAFLD by improving mitochondrial respiration and decreasing cell death [203]. FLINAX, a vitamin E-rich combination dietary supplement, has been reported to improve mitochondrial complex activity and ATP production in an HFD rat model as well as to reduce oxidative damage and hepatic steatosis [204]. In NAFLD patients, metformin, a first-line therapy for type-2 diabetes, activates AMPK and stimulates mitochondrial biogenesis and FAO [205]. Through the SIRT1/SIRT3 pathway, liraglutide, an acylated glucagon-like peptide-1 (GLP-1) agonist used as an anti-diabetic drug, has been reported to improve NAFLD in HFD mice by improving the mitochondrial architecture, reducing ROS generation, and inducing autophagy [196]. Mitotherapy, the intravenous injection of healthy mitochondria into animals with NAFLD, has been found to be associated with reduced lipid accumulation and oxidative stress in hepatocytes as well as the restoration of hepatocyte function [206]. Mitoquinone (MitoQ), a mitochondria-targeted anti-oxidant, and redox nanoparticles, which target mitochondria, are a novel class of potentially therapeutic drugs that deserve to be investigated in clinical trials [207,208]. Collectively, these data support targeting the mitochondria as a new avenue for drug development in NAFLD/NASH.

## 4. Conclusions

NAFLD is a progressive metabolic liver disease with serious complications that is rapidly becoming a major public health problem. Currently, the only available treatment modality is lifestyle modifications through diet and exercise, with limited success. To date, there are no FDA-approved drugs for its treatment. It is strongly associated with metabolic syndrome, obesity, and type 2 diabetes, and it has been shown that metabolic dysregulation is central to its pathogenesis. There is strong evidence that mitochondrial dysfunction plays a central role in development and progression of NAFLD. Studies in animal models and human subjects have linked impaired mitochondrial fatty acid oxidation and reduced mitochondrial quality to the development and progression of NAFLD. Targeting mitochondria and modulating its function is a promising new avenue for drug development to treat NAFLD/NASH.

## Data Availability

Not applicable.
